# Who perceives a higher personal risk of developing type 2 diabetes? A cross-sectional study on associations between personality traits, health-related behaviours and perceptions of susceptibility among university students in Denmark

**DOI:** 10.1186/s12889-018-5884-9

**Published:** 2018-08-04

**Authors:** Lotte Skøt, Jesper Bo Nielsen, Anja Leppin

**Affiliations:** 1 Unit for Health Promotion Research, Department of Public Health, University of Southern Denmark, Niels Bohrs Vej 9, 6700 Esbjerg, Denmark; 2 OPEN, Odense Patient Data Explorative Network, Odense University Hospital, Odense, Denmark; 3 Research Unit of General Practice, Department of Public Health, University of Southern Denmark, J.B. Winsløws Vej 9, Entrance B, 1st floor, 5000 Odense, Denmark

**Keywords:** Risk perception, Type 2 diabetes, Five-factor model, Personality traits, Health-related behaviours, Students, Health communication

## Abstract

**Background:**

Type 2 diabetes (T2D) is on the rise among young adults (aged 20–39 years). A challenge for health risk communication is that young adults may not be aware or lack acknowledgement of their personal risk of developing T2D. To date, no knowledge is available on potential relationships between personality traits and T2D risk perception in this target group. This cross-sectional study aimed to investigate direct and indirect (mediated via health-related behaviours and body mass index) associations between the Five-Factor Model personality traits and T2D risk perception among university students in Denmark.

**Methods:**

Participants included 1205 students (80% females; mean age = 25) from five major universities. All variables were assessed by means of self-report in an online questionnaire. Health-related behaviours included physical activity, sweets consumption and prior T2D screening. Covariates included socio-demographic factors and family history of T2D.

**Results:**

A hierarchical multiple regression analysis revealed that higher levels of conscientiousness and emotional stability were directly negatively associated with T2D risk perception after controlling for covariates, health-related behaviours, and body mass index. Binary logistic regression analyses showed several significant associations between personality traits and health-related behaviours as well as body mass index. Sobel tests indicated that both physical activity and body mass index partially mediated the association between conscientiousness and T2D risk perception. The association between extraversion and T2D risk perception was fully mediated by PA.

**Conclusions:**

We present novel evidence suggesting that personality traits, health-related behaviours and body mass index are associated with T2D risk perception among young adults. Thus, it may be beneficial to tailor health risk communications targeting T2D to match recipients’ personality characteristics instead of using the one size fits all approach.

## Background

Diabetes poses a major public health problem in the twenty-first Century. In Europe, the prevalence of diabetes in adults (aged 20–79) has risen from 5% in 2000 to 9.1% in 2015 [1, 2]. In Denmark, there were 320.545 children and adults with diabetes at the end of 2012, corresponding to a prevalence rate of 5.7% [3]. This is twice the number in 2002, and type 2 diabetes (T2D) accounts for approximately 80% of all reported diabetes cases [3]. Recent developments further indicate that rates of T2D are increasing among young adults (aged 20–39) [4]. T2D is a more aggressive disease when it occurs at a younger age [4, 5], particularly if it remains undetected and untreated, which may be a common occurrence among young adults since they probably are less likely to perceive themselves to be at risk because of their age. However, when people are not aware or lack acknowledgement of their personal risk, it may prevent them from taking the actions necessary to alleviate the problem or prevent it from getting worse – as outlined by common health behavior models, such as the Health Belief Model [6] or Protection Motivation Theory [7].

To inform health communications, it is important to identify the factors that determine young adults’ perceptions of T2D risk. So far, few studies have investigated T2D risk perception in this group, most of which are studies on US college students. For example, Sealey-Potts and Reyes-Velazquez [8] found an optimistic bias among students in that 68% perceived a higher T2D risk for their peers than for themselves (23%), while another study reported that optimistic bias was associated with lower scores on perceived lifetime susceptibility to T2D [9]. Mongiello and colleagues [10] compared optimistic bias towards developing T2D in students with varying risk factor load and found that even among students with three or more risk factors for T2D, 39% perceived their risk for developing T2D as being less than for other students, which likely indicates an underestimation of actual risk. As for potential determinants of young adults’ T2D risk perceptions, only family history of T2D has consistently been found to increase students’ perceived risk [10–12], whereas findings regarding socio-demographic group differences are ambiguous [10–12].

To our knowledge, research has yet to address potential relationships between personality traits and T2D risk perception. Personality traits reflect dimensions of enduring and consistent individual differences in the way people tend to think, feel, and behave [13]. Currently, the most popular taxonomy for personality traits is the Five Factor Model (FFM) [14], which comprises five basic dimensions: openness to experience (fantasy, aesthetics, feelings, actions, ideas, values), conscientiousness (competence, order, dutifulness, achievement striving, self-discipline, deliberation), extraversion (warmth, gregariousness, assertiveness, activity, excitement seeking, positive emotions), agreeableness (trust, straightforwardness, altruism, compliance, modesty, tender-mindedness), and neuroticism (sometimes named by its polar opposite, emotional stability; anxiety, angry hostility, depression, self-consciousness, impulsiveness, vulnerability). These five traits are present in varying degrees in all people [13].

The basic assumption here is that people may react to specific health risks in line with their personality characteristics. The mechanism of influence could be two-fold. First, there could be a direct influence of personality traits in that, for instance, persons who are generally more emotionally stable, more extraverted or more open to new experiences are less likely to see themselves as threatened, while those who are more emotionally unstable, more introverted or less open may generally be more prone to anticipate negative outcomes and/or experience threat. Furthermore, there may be indirect effects of personality traits mediated by health-related behaviours. People with different personality characteristics may perceive themselves as being at higher or lower risk *because* they engage in healthy or unhealthy behaviours, for instance, highly conscientious people may avoid risky health behaviours more than people low on this trait.

There is some evidence to suggest that personality traits may be directly and/or indirectly (via health-related behaviours) related to health risk perception. Sjöberg [15] found that conscientiousness and emotional stability were associated with risk perception of AIDS and unhealthy dietary habits in a sample of college applicants in Sweden. An association was also found between emotional stability and risk perception of sunrays. Another study showed that emotional stability and agreeableness were both directly and indirectly (via related health behaviours) associated with perceived susceptibility to lung cancer, alcohol dependency, and venereal disease/AIDS among university students in Switzerland [16]. Furthermore, personality traits have consistently been linked to physical activity (PA) [17, 18], dietary intake [19], and obesity [20] in diverse samples of adults − all of which are associated with T2D risk. Thus, it is possible that these factors may represent potential mediating mechanisms affecting associations between personality traits and T2D risk perception.

Given the evidence linking personality traits to health risk perception, health-related behaviours, and obesity, it is highly relevant to examine both direct and indirect pathways from personality traits to T2D risk perception via related health behaviours and body mass index (BMI) among young adults. Such research is much needed as it could help identify reasons for low T2D risk perception in this target group, which may stand in the way of preventative behavioural changes. Thus, the aim of the present study was threefold: (1) to examine associations between FFM personality traits and perceived susceptibility to T2D; (2) to explore associations between personality traits and health-related behaviours (PA, sweets consumption, prior T2D screening) and BMI; and (3) to ascertain whether potential associations between personality traits and perceived susceptibility to T2D are mediated by health-related behaviours and BMI.

## Methods

### Design

This cross-sectional study utilized the baseline data of a longitudinal (3-month follow-up) randomized controlled trial examining the persuasive effects of framed (web-based) health messages targeting the prevention of T2D. All the variables in the present study were measured in an online baseline survey. The only exception was self-reported BMI data, which was obtained immediately after the intervention. All data were collected by and stored in REDCap.^1^ Data collection began in October 2016 and ended in May 2017.

### Participants and procedure

Eligible for this study were undergraduate and postgraduate students under 40 years old attending one of five major universities in Denmark,^2^ who had more than three months left on their study program and who were not diagnosed with type 1 or type 2 diabetes.

Participants were recruited by sending an email request to study directors across the universities asking if they could forward a study announcement to students via e-mail or upload it on E-learn, social media (Facebook, Twitter) or other relevant student platforms. Interested participants could access the baseline survey in Danish or English by clicking on the weblink provided in the announcement. Upon clicking on the link, they were directed to an introductory page listing the aim of the study, eligibility criteria, methods of data collection, information about data protection, and − once consent for participation was provided − guided to the baseline survey. Upon completion of the baseline survey, participants were randomly assigned (via REDCap) to one of three groups, and subsequently (within 2–3 days) sent an email with a weblink granting access to the intervention. One group was presented with risk information on T2D directed toward young adults. The same risk information was presented to the other groups; however, these groups received additional behavioural recommendations highlighting the costs of failing to adhere to the recommendations (loss frame) or the benefits of adhering to the recommendations (gain frame). After reading the health brochure, participants completed a series of post measures. Three months later, participants were sent an email with a weblink granting access to the follow-up survey.

### Measures

#### Baseline survey

Perceived (lifetime) susceptibility to T2D was assessed using a single Likert-type item: “How likely do you think it is that you will develop type 2 diabetes at some point in your life?” Responses were rated on a 7-point scale ranging from 1 = extremely unlikely to 7 = almost certain.

Covariates included age, sex, parental education, parental birthplace^3^ and family history of T2D (immediate or extended family members). Responses on the following items were collapsed into two categories: parental education (university vs. other), parental birthplace (both parents born in Denmark vs. other) and family history of T2D (no/don’t know vs. yes). Participants who responded ‘I don’t know’ to parental education and/or parental birthplace were coded as missing.

Moderate and vigorous PA were assessed separately by asking: “In the last three months, how many times a week on average did you engage in moderate-intensity physical activity for at least 30 minutes a day/vigorous-intensity physical activity for at least 20-30 minutes a day?” The response options were: ‘less than once a week’, ‘once a week’, 2 times a week’, ‘3-4 times a week’, ‘5-6 times a week’, ‘7 times a week’. Cut-off points were based on the Danish Health Authority’s recommendations for weekly physical activity for adults aged 18–64 years: ‘7 times a week’ for moderate PA, and ‘more than once a week’ for vigorous PA [21]. Scores on both indicators were combined to for a composite PA variable with two categories: 'below recommended level' (below cut-off on both moderate and vigorous PA) vs. 'at or above recommended level' (above or equal to the cut-off on either moderate or vigorous PA).

Sweets consumption was assessed by asking: In the last three months, how often on average did you eat sweets (chocolate, cookies, winegums etc.) and/or ice cream? The response options were: ‘less than once a month’, ‘once a month’, ‘several times a month’, ‘once a week’, ‘several times a week’, ‘once a day’, ‘more than once a day’. The variable was dichotomized into: ‘less than once a day’ vs. ‘once a day or more’.

Prior T2D screening was assessed by asking: Have you ever been tested for type 2 diabetes (yes or no)?

Personality traits were measured using the Ten-Item Personality Inventory (TIPI) [22]; a widely used, brief measure of the FFM personality traits, applicable in situations where the comprehensive gold-standard assessment (NEO-PI-3) [23] cannot be used. The TIPI has two items per dimension consisting of pairs of adjectives, each measuring the same pole of one of the FFM dimensions. Half of the items represent the positive pole of the construct and the other half the negative pole. Each item is rated on a 7-point Likert scale ranging from 1 = disagree strongly to 7 = agree strongly. A total score for each dimension is generated by inversing the reverse-scored item, and then computing the average of the two. Higher scores indicate higher levels of that particular personality trait. Since no Danish version of the TIPI exists we arranged for the scale to be translated to Danish and then back-translated to English (please contact the authors for the full procedure). In the present study, we used both the original TIPI and the translated Danish version.

#### Post-intervention survey

Participants in all three groups of the trial were asked to complete a series of postmeasures (not relevant to this study) directly after having read the respective health brochures. They were also requested to report their weight and height, which was used to calculate BMI (kg/m^2^). BMI was dichotomized into: underweight/normal weight (BMI < 25) vs. overweight/obese (BMI ≥25), consistent with the World Health Organization’s (WHO) BMI classification [24]. In the present study, BMI was treated as a baseline variable since scores were not expected to undergo relevant changes between baseline and post-intervention.

### Statistical analysis

The continuous variables, which included age, personality traits, and perceived susceptibility to T2D, were described using the mean and standard deviation. Since standard significance tests for normality, such as Kolmogorov-Smirnov and Shapiro−Wilks are known to be overly sensitive to large samples, we relied on visual inspection of histograms and normal q-q plots as well as p-p plots of the standardized residuals, which indicated no severe deviations from normality.

Dichotomous variables were described using frequency counts and percentages. Participants with missing data on one or more of the variables of interest were excluded from the analyses. The Independent samples t-test or chi square tests were used to establish whether sample characteristics (used as covariates) differed between participants with complete and incomplete data. Pearson correlation coefficients were computed to examine (two-tailed) bivariate associations between potential predictors and perceived susceptibility to T2D.

The Baron and Kenny (1989) approach was used to test for mediation [25]. First, a hierarchical linear multiple regression analysis controlling for covariates was conducted to test: (1) the effects of personality traits on perceived susceptibility to T2D in the absence of potential mediators, and (2) the unique effects of personality traits, health-related behaviours and BMI on perceived susceptibility to T2D. Covariates and personality traits were entered on the first step, and health-related behaviours and BMI on the second step. Next, a series of binary logistic regression analyses controlling for covariates were performed to examine the effects of personality traits on BMI (model 1), composite PA (model 2), sweets consumption (model 3), and prior T2D screening (model 4). Finally, the Sobel test [26] was used to test the significance of any mediation effects.

All analyses were performed using IBM SPSS Statistics for Windows, Version 24.0 (Armonk, NY: IBM Corp). *P*-values less than .05 were considered as statistically significant.

## Results

The baseline survey was accessed 2157 times. A total of 607 individuals were excluded from the study due to: declining to participate (*n* = 151), responding to the questions in a systematic fashion (*n* = 2), not meeting the age criteria (*n* = 101), dropping out of the survey (*n* = 329), or submitting the survey with missing responses (*n* = 24). The remaining 1550 participants were randomly allocated to the intervention groups. After randomization, a further 344 participants were excluded for either dropping out of the intervention or submitting the post-intervention survey with missing BMI data. One participant represented a multivariate outlier and was subsequently excluded. The final study sample included 1205 participants; corresponding to 60% of those who filled out the baseline survey (*n* = 2006).

There were no significant differences in age [t (1899) = 1.772, *p* = .077), sex [χ^2^ = 1.482, *p* = .223], parental education [χ^2^ = 1.418, *p* = .234], or family history of T2D [χ^2^ = .591, *p* = .442] between participants who provided complete (*n* = 1205) and incomplete (*n* = 697) data. Parental birthplace was not equally distributed across the two groups [χ^2^ = 10.793, *p* = .001]. Compared to participants with incomplete data, a higher proportion of those with complete data reported having both parents born in Denmark.

### Sample characteristics

Of the 1117 participants who responded to the question about which faculty they were enrolled in, 374 (33.5%) represented Humanities; 240 (21.5%) Natural Sciences, 223 (20%) Health Sciences or Medicine; 222 (19.9%) Social Sciences and/or Business; and 51 (4.5%) Engineering. The remaining 7 (0.6%) participants represented other faculties. As shown in Table 1, participants in the final study sample (*n* = 1205) were predominantly female (80.2%) and 25 years old on average. The proportions of students enrolled in undergraduate (47.8%) and postgraduate (52.2%) degree programs were roughly equal. The large majority (82.3%) reported having both parents born in Denmark. Over half (57%) had at least one parent with a university education. Approximately one third (29.6%) had a family history of T2D. A quarter (25%) were overweight/obese, while only a small minority (12.1%) had been screened for T2D in the past. Over half (54.4%) reached officially recommended levels of PA, and the clear majority (90.4%) reported eating sweets less than once a day.

**Table 1:** Characteristics of the study sample (*n* = 1205)

Variables	Categories	N (%) or Mean (Range; ±SD)
Degree program	Undergraduate	576 (47.8)
	Postgraduate	629 (52.2)
Age		24.9 (19–39; ±4.0)
Sex	Male	238 (19.8)
	Female	967 (80.2)
Parental education	Other	518 (43)
	University	687 (57)
Parental birthplace	Other	213 (17.7)
	Both parents born in Denmark	992 (82.3)
Family history of T2D	No/don’t know	848 (70.4)
	Yes	357 (29.6)
BMI	Underweight/normal weight	904 (75)
	Overweight/Obese	301 (25)
Prior T2D screening	No	1059 (87.9)
	Yes	146 (12.1)
Composite PA	At or above recommended level	656 (54.4)
	Below recommended level	549 (45.6)
Sweet consumption	Less than once a day	1089 (90.4)
	Once a day or more	116 (9.6)
Openness to experience		5.0 (1–7; ±1.1)
Conscientiousness		5.4 (1–7; ±1.2)
Extraversion		4.3 (1–7; ±1.6)
Agreeableness		4.8 (1–7; ±1.1)
Emotional stability		4.6 (1–7; ±1.4)
Perceived susceptibility to T2D		2.72 (1–7; ±1.2)

### Bivariate analyses

The results of the correlational analyses between potential predictors and perceived susceptibility to T2D (a correlation table is available from the authors) were as follows. Higher perceived susceptibility scores were significantly associated with having a family history of T2D (*r* = .227) being overweight/obese (*r* = .230), having been screened for T2D in the past (*r* = .129), and eating sweets once a day or more (*r* = .092). Lower perceived susceptibility scores were significantly associated with having at least one parent with a university education (*r* = −.090), meeting recommended levels of PA (*r* = −.197) as well as the personality traits of openness (*r* = −.085), conscientiousness (*r* = −.246), extraversion (*r* = −.116), and emotional stability (*r* = −.190). All correlations were significant at the 1% level.

### Regression analyses

Table 3 presents the results of the hierarchical linear multiple regression analysis predicting perceived susceptibility to T2D. In step 1, the variance accounted for by the covariates and personality traits was 13.5%, which was significantly different from zero. Of the covariates, parental education and family history of T2D were the only significant predictors. Conscientiousness, extraversion and emotional stability were significantly negatively associated with perceived susceptibility scores. The addition of health-related behaviours and BMI in step 2 significantly increased the amount of explained variance from 13.5 to 19.3%. BMI, sweets consumption, and prior T2D screening were significantly positively associated with perceived susceptibility scores, whereas composite PA showed a negative association. The addition of health-related behaviours and BMI in step 2 slightly reduced the regression coefficients for conscientiousness, extraversion and emotional stability. Conscientiousness and emotional stability, but not extraversion, remained significant predictors along with parental education and family history of T2D.

Table 2 presents the results of the binary logistic regression analyses predicting health-related behaviours and BMI. After controlling for covariates, lower levels of conscientiousness and openness were significantly associated with being overweight/obese. Higher levels of conscientiousness, extraversion and emotional stability were significantly associated with meeting recommended levels of PA. Lower extraversion levels were significantly associated with eating sweets once a day or more, and higher levels of openness with having been screened for T2D in the past.

### Establishing mediation

The results of the mediation analysis can be summarized as follows:

1. Conscientiousness, extraversion, and emotional stability were significantly negatively associated with perceived susceptibility to T2D in the absence of health-related behaviours and BMI (Table 2).
2. Conscientiousness and emotional stability, but not extraversion, continued to be significantly associated with perceived susceptibility to T2D after controlling for health-related behaviours and BMI (Table 2).
3. Conscientiousness was associated with PA and BMI, extraversion with PA and sweets consumption, and emotional stability with PA (Table 3).

**Table 2:** Hierarchical linear multiple regression analysis predicting perceived susceptibility to T2D (*n* = 1205)

Predictors	Step 1^a^				Step 2^b^			
	B	95% CI	β	*p*	B	95% CI	β	*p*
(Constant)	4.498	3.896–5.099		.000	4.534	3.949–5.120		.000
Age	.000	−.016–.015	−.001	.971	−.012	−.027–.003	−.042	.118
Gender 0 = Male; 1 = Female	.013	−.151–.177	.004	.875	−.016	−.175–.144	−.005	.847
Parental education 0 = Other; 1 = University	−.179	−.304–−.054	−.076	.005*	−.138	−.259–−.017	−.058	.026*
Parental birthplace 0 = Other; 1 = Both parents born in DK	−.062	−.225–.101	−.020	.458	−.028	−.186–.130	−.009	.727
Family history of T2D 0 = No/Don’t know; 1 = Yes	.516	.381–.651	.202	.000**	.440	.308–.572	.172	.000**
Openness	−.056	−.112–.001	−.054	.054	−.055	−.110–.000	−.053	.052
Conscientiousness	−.202	−.253–−.150	−.214	.000**	−.172	−.222–−.122	−.182	.000**
Extraversion	−.044	−.084–−.004	−.061	.032*	−.035	−.074–.004	−.049	.076
Agreeableness	.057	−.022–.116	.053	.057	.051	−.006–.108	.047	.082
Emotional stability	−.106	−.153–−.058	−.127	.000**	−.097	−.143–−.050	−.116	.000**
BMI 0 = Underweight/normal weight; 1 = Overweight/obese					.469	.328–.609	.174	.000**
Prior T2D screening 0 = No; 1 = Yes					.257	.070–.445	.072	.007*
Composite PA 0 = Below recommended level; 1 = At or above recommended level					−.298	−.420–−.176	−.127	.000**
Sweets consumption 0 = Less than once a day; 1 = Once a day or more					.268	.063–.472	.068	.010*

^a^Step 1: adj. *R*^2^ = .135, F^change^ (10, 1194) = 19.77, *p* < .001; ^b^Step 2: adj. *R*^2^ = .193, F^change^ (4, 1190) = 22.66, *p* < .001

*p < .05; **p < .001

**Table 3:** Binary logistic regression analyses predicting health-related behaviours and BMI (*n* = 1205)

Predictors	Model 1: BMI				Model 2: Composite PA				Model 3: Sweets consumption				Model 4: Prior T2D screening			
	Wald	OR	95% CI	*p*	Wald	OR	95% CI	*p*	Wald	OR	95% CI	*p*	Wald	OR	95% CI	*p*
Age	8.910	1.050	1.017–1.084	.003*	6.070	.963	.935–.992	.014*	16.703	1.094	1.048–1.141	.000**	17.185	1.089	1.046−1.133	.000**
Sex 0 = Male 1 = Female	1.560	1.245	.883–1.754	.212	4.251	1.385	1.016–1.887	.039*	6.644	.452	.247–.827	.010*	2.112	.683	.408–1.142	.146
Parental education 0 = Other 1 = University	5.000	1.357	1.038–1.774	.025*	1.194	.877	.692–1.110	.275	.138	1.078	.726–1.600	.710	.730	1.170	.816–1.676	.383
Parental birthplace 0 = Other 1 = Both parents born in Denmark	.168	1.076	.759–1.524	.682	1.229	.838	.614–1.145	.268	6.803	1.841	1.164–2.911	.009*	.084	.933	.584–1.491	.773
Family history of T2D 0 = No/don’t know 1 = Yes	8.057	.665	.502–.882	.005*	3.103	1.259	.974–1.628	.078	.179	1.098	.713–1.690	.672	22.159	.421	.293–.603	.000**
Openness	4.135	.882	.782–.995	.044*	1.882	.928	.833–1.033	.170	.286	.953	.797–1.138	.593	6.123	1.236	1.045−1.461	.013*
Conscientiousness	6.112	.871	.781–.972	.013*	14.660	1.215	1.100–1.342	.000**	3.610	.860	.736–1.005	.057	.096	.977	.842–1.133	.756
Extraversion	.159	1.018	.933–1.110	.690	8.366	1.119	1.037–1.208	.004*	4.257	.875	.771–.993	.039*	.390	1.038	.924–1.165	.532
Agreeableness	1.895	1.094	.963–1.244	.169	1.299	.937	.838–1.048	.254	.868	.913	.755–1.105	.351	2.873	.860	.722–1.024	.090
Emotional stability	.001	.998	.901–1.107	.975	3.899	1.095	1.001–1.199	.048*	.094	.977	.841–1.135	.759	.767	.941	.821–1.079	.381

*p < .05; **p < .001

The results of the Sobel tests revealed that both PA [z = − 2.992, *p* < .01] and BMI [z = − 2.304, *p* = .02] mediated the association between conscientiousness and perceived susceptibility to T2D. Furthermore, the association between extraversion and perceived susceptibility to T2D was mediated by PA [z = − 2.481, *p* = .01], but not sweets consumption [z = 1.609, *p* = .11]. Finally, there was no mediation effect of PA on the association between emotional stability and perceived susceptibility to T2D [z = − 1.829, *p* = .07].

## Discussion

This study is unique in its examination of associations between FFM personality traits, health-related behaviours, BMI and perceived susceptibility to T2D among university students. Our results revealed that conscientiousness and emotional stability were directly negatively associated with T2D risk perception after controlling for socio-demographic factors, family history of T2D, health-related behaviours and BMI. Also, conscientiousness was found to be associated with PA and BMI, extraversion with PA and sweets consumption, emotional stability with PA, and openness with BMI and prior T2D screening. Furthermore, both PA and BMI partially mediated the association between conscientiousness and T2D risk perception. The association between extraversion and T2D risk perception was fully mediated by PA.

The results regarding T2D risk perception indicated that, on average, students did not perceive themselves to be particularly susceptible to developing T2D during their lifetime (mean = 2.72 on a scale from 1 to 7), which is consistent with prior research [8–10]. As for the role of FFM personality traits in T2D risk perception, students high on conscientiousness or emotional stability were more optimistic regarding their future T2D risk. Vollrath and colleagues [16] found similar associations in a sample of university students in Switzerland. This tendency may be rooted in the competent, dutiful and self-disciplined nature of conscientious personalities making them well-equipped to deal with life and less likely to take risks with their health, while the calm, relaxed nature of emotionally stable personalities may make them less likely to dwell on things that may go wrong.

Our finding that higher levels of conscientiousness, extraversion and emotional stability were related to increased PA is consistent with the results of previous meta-analyses [17, 18]. However, while these meta-analyses also report a positive association between openness and PA, we found no such association. Participants in the studies included in the meta-analyses were generally older than the students in the present study, and since PA is more common among younger adults [27], it may be that students do not need to be particularly open to new experiences to be exposed to PA, or vice versa they may experience PA as considerably less novel than older adults.

In this study, higher levels of extraversion were found to be protective in terms of sweets consumption. This contrasts with a previous Swiss population study [28], which showed that high extraversion promoted sweets consumption via the tendency to eat in response to external cues present (external eating). It is possible that sweets consumption may be less involved in the social activities sought out by extraverted students (e.g. sports activities, visiting pubs/discos) than in those that attract older extraverted adults in the general population (e.g. family gatherings). While weight concerns may in general have been highly relevant for students given that most were female and under 30 years old, this tendency may have been even stronger among the highly extraverted who are particularly interested in creating favourable social impressions or images. As for T2D screening, to our knowledge, no studies have yet investigated the role of personality traits in T2D screening. However, based on our results, it is plausible that those who are generally more open to new experiences are also more curious to participate in health screenings.

Consistent with the results of a systematic review conducted by Gerlach and colleagues [20], we found that higher levels of conscientiousness were protective against overweight/obesity. Contrary to the Gerlach et al. review, which showed a positive association between neuroticism (emotional stability) and overweight/obesity, we found no association between emotional stability and BMI. Moreover, while we identified a negative association between openness and BMI, Gerlach and colleagues found no evidence for the role of openness in body weight. The discrepancy in findings may be due to age differences between study samples. Most of the studies in the review included adult samples with mean ages in the midlife period (40−50 years). At this stage in life, obesity status is likely to have stabilized substantially. Thus, it is possible that personality traits may exhibit different patterns with body weight in young adulthood.

Similar to the study by Vollrath and colleagues [16], which found that conscientiousness had negative indirect effects on perceptions of susceptibility to health risks via related health behaviours, our study showed that both PA and BMI partially mediated the association between conscientiousness and T2D risk perception. Previous research based on follow-up data from the Terman life-cycle study, which followed 1253 US Americans over 7 decades, found that personality traits, particularly conscientiousness, negatively predicted health behaviours (alcohol use and smoking) and BMI across the full lifespan (1930–2000) [29]. This indicates that conscientious individuals are more likely to do the right things for their health. In line with this research, our results suggest that conscientious students were more likely to reach recommended levels of PA and maintain healthy BMIs because they know that these are the right things to do to prevent adverse health outcomes, which in turn, has lessened their perceptions of T2D risk.

Whereas Vollrath and colleagues found no evidence for the role of extraversion in predicting perceptions of susceptibility to health risks, our results showed that PA fully mediated the association between extraversion and T2D risk perception. This suggests that extraversion went along with a higher likelihood to reach recommended levels of PA, which again coincided with lesser T2D risk perception. One possible explanation for this finding is that extraverted students may be part of a social group which expects people to partake in PA, and, given that extraverted people are interested in creating favourable social impressions or images, this may have motivated them to engage in regular PA. Furthermore, they may also have knowledge about preventive behaviours for T2D, which could possibly have influenced their T2D risk perception.

A strength of the present study lies in the large sample of students representing five major universities in Denmark, but there are also some limitations. Since this study was cross-sectional, we cannot claim causality. However, based on theory one can argue that it is highly implausible to assume that personality traits, which are conceived of as stable over time [13], should be predicted by T2D risk perception, health-related behaviours, or BMI. On the other hand, it is more difficult to establish causality regarding the association between health-related behaviours and risk perception. In this case, we were interested in investigating a potential influence of prior behaviour on risk perception, while common health behaviour theories, such as the Health Belief Model [6] and Protection Motivation Theory [7] claim that health-related behaviours are predicted by risk perception. Such assumptions are, however, not mutually exclusive, since causality most likely is inherently bi-directional. People’s risk perceptions may influence behavioural change towards more health-protective behaviour, just as health behaviour models predict. However, when assessing their own risk; people will also consider their prior level of health-protective or health-detrimental behaviours [30–33]. Further prospective research is warranted to address the question of whether health-related behaviours and BMI play a role in shaping T2D risk perceptions.

Caution must be taken in generalizing the findings of this work, because convenience sampling was used. This yielded a sample of predominantly female students, which though common in student surveys [34], is bound to reflect the higher interest in and concerns about health issues among women. In general, it must be assumed that it was mainly students interested in health topics who were willing to participate in the first place and less likely to drop out of the survey. This is likely to have introduced sampling bias to the study. The fact that BMI was measured after the intervention, and not at baseline, has resulted in some further loss of data, which may have increased this bias towards persons interested in health issues. Furthermore, we could not establish whether there was congruence between students’ actual and perceived T2D risk because there was insufficient data to calculate actual T2D risk. However, we did adjust for some of the risk factors for T2D, such as socio-demographic factors, family history of T2D, and BMI.

Another potentially limiting factor is that some recall bias and social desirability may have occurred since all variables were measured by means of self-report. The BMIs utilized in this study may not have been precise given evidence that males tend to overestimate their height while females tend to underestimate their weight [35]. Furthermore, we only measured frequency of sweets consumption because it is more demanding for participants to provide information about portion sizes. This has resulted in the loss of some resolution about sweets consumption. Having information about portion sizes may have resulted in more differentiation and possibly also stronger associations for this variable. A final limitation is that, due to time constraints, we opted to use a brief measure of the FFM personality traits (TIPI), rather than the comprehensive gold standard (NEO-PI-3). However, the TIPI has been shown to have adequate levels of convergent and discriminant validity, as well as test-retest reliability [20]. Moreover, Ehrhart and colleagues [36] reported favourable outcomes in terms of the factor structure and convergent validity of the TIPI.

## Conclusion

Although our findings need further confirmation, particularly by longitudinal studies as well as studies systematically testing the effects of health risk messages, the present research demonstrates the utility of personality traits in understanding T2D risk perception among young adults. We confirmed earlier analyses showing that students have low T2D risk perception, and we extended them by demonstrating direct and indirect (via health-related behaviours and BMI) associations between FFM personality traits and T2D risk perception. One practical implication of these findings is that it may be beneficial to tailor health risk communications to match recipients’ personality characteristics instead of using the one size fits all approach. Support for the tailored approach is provided by Hirsh and colleagues [37] who found that survey respondents evaluated tailored advertisements for a single product more positively the more they cohered with their personality characteristics. There is also evidence to suggest that there is some promise to using personality traits as a method for adapting health-promoting mobile applications to better fit the needs of target audiences [38]. An important objective of future research is to examine the effectiveness of using this technique in persuasive health risk communications targeting early onset T2D.

## Abbreviations

BMI: Body mass index
CI: Confidence interval
FFM: Five-factor model
PA: Physical activity
SD: Standard deviation
T2D: Type 2 diabetes
TIPI: Ten item personality inventory

## Footnotes

1 Research Electronic Data Capture

2 Aarhus University, University of Copenhagen, Aalborg University, University of Southern Denmark, and Roskilde University

3 Proxy measure for having a Danish vs. foreign/immigrant background

## References

[1] International Diabetes Federation (2000). IDF Diabetes Atlas.

[2] International Diabetes Federation (2015). IDF Diabetes Atlas.

[3] Danish Diabetes Association. Diabetes i Danmark. 2018. Retrived from https://diabetes.dk/presse/diabetes-i-tal/diabetes-i-danmark. Accessed 2 Jan 2018.

[4] Lascar N, Brown J, Pattison H, Barnett AH, Bailey CJ, Bellary S (2017). Type 2 diabetes in adolescents and young adults. Lancet Diabetes Endocrinol.

[5] Song SH, Hardisty CA (2008). Early-onset type 2 diabetes mellitus: an increasing phenomenon of elevated cardiovascular risk. Expert Rev Cardiovasc Ther.

[6] Janz NK, Becker MH (1984). The health belief model: a decade later. Health Educ Q.

[7] Rogers RW, Cacioppo J, Petty R (1983). Cognitive and physiological processes in fear appeals and attitude change: a revised theory of protection motivation. Social psychophysiology.

[8] Sealey-Potts C, Reyes-Velazquez W (2014). Perceived and actual risks of college students for developing type 2 diabetes. Austin J Nutr Metab.

[9] Reyes-Velazquez W, Sealey-Potts C (2015). Unrealistic optimism, sex, and risk perception of type 2 diabetes onset: implications for education programs. Diabetes Spectr.

[10] Mongiello LL, Freudenberg N, Jones H, Spark A (2016). Many college students underestimate diabetes risk. J Allied Health.

[11] Dickerson JB, Smith ML, Sosa E, McKyer EL, Ory MG (2012). Perceived risk of developing diabetes in early adulthood: beliefs about inherited and behavioral risk factors across the life course. J Health Psychol.

[12] Dong-Chul S, Torabi MR, Kaigang L, John PM, Woodcox SG, Perera B (2008). Perceived susceptibility to diabetes and attitudes owards preventing diabetes among college students at a large midwestern university. Am J Health Stud.

[13] McCrae RR, Costa PT (2006). Personality in adulthood: a five factor theory perspective.

[14] McCrae RR, Costa PT (1997). Personality trait structure as a human universal. Am Psychol.

[15] Sjöberg L (2003). Distal factors in risk perception. J Risk Res.

[16] Vollrath M, Knoch D, Cassano L. Personality risky behaviour, and perceived susceptibility to health risks. Eur J Pers. 1999;13(1):39–50.

[17] Wilson KE, Dishman RK (2015). Personality and physical activity: a systematic review and meta-analysis. Personal Individ Differ.

[18] Sutin AR, Stephan Y, Luchetti M, Artese A, Oshio A, Terracciano A (2016). The five-factor model of personality and physical inactivity: a meta-analysis of 16 samples. J Res Pers.

[19] Lunn TE, Nowson CA, Worsley A, Torres SJ (2014). Does personality affect dietary intake?. Nutrition.

[20] Gerlach G, Herpertz S, Loeber S (2015). Personality traits and obesity: a systematic review. Obes Rev.

[21] Sundhedsstyrrelsen. Fysisk aktivitet - håndbog om forebyggelse og behandling. Rosendahls-Schultz A/S; 2011.

[22] Gosling SD, Rentfrow PJ, Swann WB (2003). A very brief measure of the big-five personality domains. J Res Pers.

[23] McCrae RR, Costa PT, Martin TA (2005). The NEO-PI-3: a more readable revised NEO personality inventory. J Pers Assess.

[24] World Health Organization (2018). BMI classification.

[25] Baron RM, Kenny DA (1986). The moderator-mediator variable distinction in social psychological research: conceptual, strategic, and statistical considerations. J Pers Soc Psychol.

[26] Sobel ME (1982). Asymptotic confidence intervals for indirect effects in structural equation models. Sociol Methodol.

[27] Bauman AE, Reis RS, Sallis JF, Wells JC, Loos RJF, Martin BW (2012). Correlates of physical activity: why are some people physically active and others not?. Lancet.

[28] Keller C, Siegrist M (2015). Does personality influence eating styles and food choices? Direct and indirect effects. Appetite.

[29] Martin LR, Friedman HS, Schwartz JE (2007). Personality and mortality risk across the life span: the importance of conscientiousness as a biopsychosocial attribute. Health Psychol.

[30] Leppin A, Aro AR (2009). Risk perceptions related to SARS and avian influenza: theoretical foundations of current empirical research. Int J Behav Med.

[31] Brewer NT, Chapman GB, Gibbons FX, Gerrard M, McCaul KD, Weinstein ND (2007). Meta-analysis of the relationship between risk perception and health behavior: the example of vaccination. Health Psychol.

[32] Brewer NT, Weinstein ND, Cuite CL, Herrington JE (2004). Risk perceptions and their relation to risk behavior. Ann Behav Med.

[33] Siegrist M (2013). The necessity for longitudinal studies in risk perception research. Risk Anal.

[34] Porter SR, Whitcomb ME (2005). Non-response in student surveys: the role of demographics, engagement and personality. Res High Educ.

[35] Stommel M, Schoenborn CA (2009). Accuracy and usefulness of BMI measures based on self-reported weight and height: findings from the NHANES & NHIS 2001-2006. BMC Public Health.

[36] Ehrhart MG, Ehrhart KH, Roesch SC, Chung-Herrera BG, Nadler K, Bradshaw K (2009). Testing the latent factor structure and construct validity of the ten-item personality inventory. Personal Individ Differ.

[37] Hirsh JB, Kang SK, Bodenhausen GV (2012). Personalized persuasion: tailoring persuasive appeals to recipients’ personality traits. Psychol Sci.

[38] Halko S, Kientz JA. Personality and persuasive technology: an exploratory study on health-promoting mobile applications. In: Persuasive technology. Berlin: Springer Berlin Heidelberg. 2010. p 150–61.

